# The potential of muscarinic M_1_ and M_4_ receptor activators for the treatment of cognitive impairment associated with schizophrenia

**DOI:** 10.3389/fpsyt.2024.1421554

**Published:** 2024-10-04

**Authors:** Samantha E. Yohn, Phillip D. Harvey, Stephen K. Brannan, William P. Horan

**Affiliations:** ^1^ Bristol Myers Squibb, Princeton, NJ, United States; ^2^ Division of Psychology, University of Miami, Miami, FL, United States; ^3^ Department of Psychiatry & Biobehavioral Sciences, University of California, Los Angeles, Los Angeles, CA, United States

**Keywords:** muscarinic, cognition, acetylcholine, cognitive impairment associated with schizophrenia, schizophrenia, M1 receptor, M4 receptor

## Abstract

Cognitive impairment is a core symptom of schizophrenia and a major determinant of poor long-term functional outcomes. Despite considerable efforts, we do not yet have any approved pharmacological treatments for cognitive impairment associated with schizophrenia (CIAS). A combination of advances in pre-clinical research and recent clinical trial findings have led to a resurgence of interest in the cognition-enhancing potential of novel muscarinic acetylcholine receptor (mAChR) agonists in schizophrenia. This article provides an overview of the scientific rationale for targeting M_1_ and M_4_ mAChRs. We describe the evolution of neuroscience research on these receptors since early drug discovery efforts focused on the mAChR agonist xanomeline. This work has revealed that M_1_ and M_4_ mAChRs are highly expressed in brain regions that are implicated in cognition. The functional significance of M_1_ and M_4_ mAChRs has been extensively characterized in animal models via use of selective receptor subtype compounds through neuronal and non-neuronal mechanisms. Recent clinical trials of a dual M_1_/M_4_ mAChR agonist show promising, replicable evidence of potential pro-cognitive effects in schizophrenia, with several other mAChR agonists in clinical development.

## 1 Introduction

Schizophrenia is a complex, heterogenous psychiatric disorder characterized by an array of debilitating symptoms and one of the top 10 leading causes of disability worldwide (1). Symptoms of schizophrenia span three main domains: positive, negative and cognitive symptoms, which vary in relative severity between affected individuals (1). Positive symptoms include delusions and hallucinations as well as disorganized speech/behavior. These symptoms generally respond well to antipsychotic medications though many patients continue to experience residual symptoms and adverse side effects (e.g., weight gain, metabolic disturbances) (1, 2). Negative symptoms include social withdrawal, lack of motivation, anhedonia, and flattened affect. These symptoms typically do not respond to antipsychotic medications and contribute to chronic functional disability for many patients (3).

This review focuses on the cognitive symptoms of schizophrenia. Since the earliest description of schizophrenia by Kraeplin as “dementia praecox,” cognitive deficits have been considered a core component of this debilitating neuropsychiatric disorder (4, 5). Schizophrenia is characterized by broad impairment across multiple cognitive domains, such as learning and memory, reasoning and problem solving, speed of processing, and attention (6). The magnitude of impairment is substantial, with people with schizophrenia, on average, falling 1.5 to 2 standard deviations (SD) below healthy normative standards (7, 8). Cognitive impairment associated with schizophrenia (CIAS) is distinct and separate from positive and negative symptoms, is present prior to the initial onset of positive symptoms, and highly stable across both symptom state changes and the longitudinal course of illness (9). Importantly, like negative symptoms, CIAS is a major contributor to poor long−term functional outcomes, impeding the ability of people with schizophrenia to live independently, attain competitive employment, and develop supportive social networks (10, 11).

No efficacious pharmacological treatments for CIAS yet exist. Approved first-line treatments for schizophrenia, including the first- and second-generation antipsychotics that rely on D_2_ dopamine (DA) receptor blockade, do not meaningfully impact cognitive deficits (12–14). The National Institute of Mental Health developed the Measurement and Treatment Research to Improve Cognition in Schizophrenia (MATRICS) initiative in the early 2000s, which stimulated major efforts to develop novel adjunctive agents, co-administered with an antipsychotic, for CIAS. Based on their role in healthy cognition and in the pathophysiology of cognitive impairments in schizophrenia, these efforts have focused on regulating four neurotransmitter systems.

• Cortical DA signaling plays a central role in normal attention, working memory, and executive functions as well as inhibiting unrelated noise to fine-tune adaptive neural signaling. In schizophrenia, DA dysregulation is strongly linked to positive symptoms via presynaptic hyperdopaminergia in striatal regions as well as cognitive impairment via cortical hypodopaminergia (15).
• Balanced and coordinated activity between excitatory excitatory glutamate (Glu) pyramidal cells and inhibitory Gamma-aminobutyric acid (GABA) interneurons is essential for normal learning, working memory, and neuroplasticity. In schizophrenia, cortical disinhibition associated with an altered excitatory and inhibitory balance between these neurotransmitters is thought to produce discoordination in neural networks that results in cognitive deficits (16).
• The two families of Acetylcholine (ACh) receptors, nicotinic (nAChRs) and muscarinic (mAChRs), are both associated with a range of cognitive functions, including learning, sensory gating, episodic memory, working memory, spatial memory, and attention (17). In schizophrenia, most treatment development has focused on the nAChR system. Impairment in several cognitive domains has been linked to nAChRs, particularly the α7 subunit (18).

Unfortunately, dozens of trials aimed at enhancing or restoring DA, Glu and GABA, or ACh via nAChRs for CIAS through adjunctive treatments have been plagued by replication failures and have not led to any regulatory approvals (13). There remains a very significant unmet need for more efficacious treatments for cognitive impairment based on new mechanisms and modes of action.

Despite the discouraging history, there has been a recent surge of interest in and optimism about CIAS drug development efforts focused on mAChRs. This renewed excitement comes from trials indicating that M_1_ and M_4_ mAChR-targeted drugs, which do not have direct antagonist effects on D_2_ DA receptors, can effectively treat not only the positive symptoms of schizophrenia but possibly cognitive impairments, as well. This article describes the evolution of clinical and pre-clinical *in vivo* and *ex vivo* research that supports this promising, though not entirely new, treatment approach for CIAS. First, the story traces its origins to early clinical drug discovery efforts in the 1990s that were searching for ACh-targeted treatments to impact the cognitive impairment associated with Alzheimer’s disease (AD). Second, these clinical findings motivated decades of basic neuroscience research on M_1_ and M_4_ mAChRs, which has extensively characterized their distribution and function (e.g., neuronal and non-neuronal) in brain regions implicated in cognition via *in vitro* assays and animal models. Finally, current clinical research in schizophrenia in the past 5 years has demonstrated the impact of M_1_ and M_4_ mAChR activators as an entirely novel monotherapy approach for multiple symptom domains of schizophrenia, potentially including cognitive impairment.

## 2 Early clinical drug discovery efforts

In the 1990s, early clinical efforts to evaluate the therapeutic potential of ACh-modulating drugs focused on discovering new treatments for cognitive impairment in AD. Evidence that AD is associated with a degeneration of cholinergic neurons motivated efforts to pharmacologically enhance cholinergic signaling. Initial efforts to broadly improve cholinergic transmission and enhance cognitive function in AD focused on acetylcholinesterase (AChE) inhibitors (e.g., tacrine, physostigmine, and donepezil; for detailed review, see (19). Although AChE inhibitors are still used today for symptom treatment, their efficacy is modest at best (20). During the same period, parallel pre-clinical research (e.g., cell-based assays and animal models) led to a much deeper and refined understanding of the cholinergic system.

### 2.1 Muscarinic cholinergic system

The cholinergic system is one of the most important modulatory neurotransmitter systems in the brain as it controls a wide range of activities (21). Of relevance to the pathophysiology of schizophrenia, cholinergic innervation can be split into two primary networks: the hindbrain complex that projects to the midbrain, which plays an important modulatory role in neural circuits implicated in psychosis [see (22) for review], and the forebrain complex, which projects to the cortical regions involved in cognitive function (e.g., frontal cortex and hippocampus).

**Figure 1:**
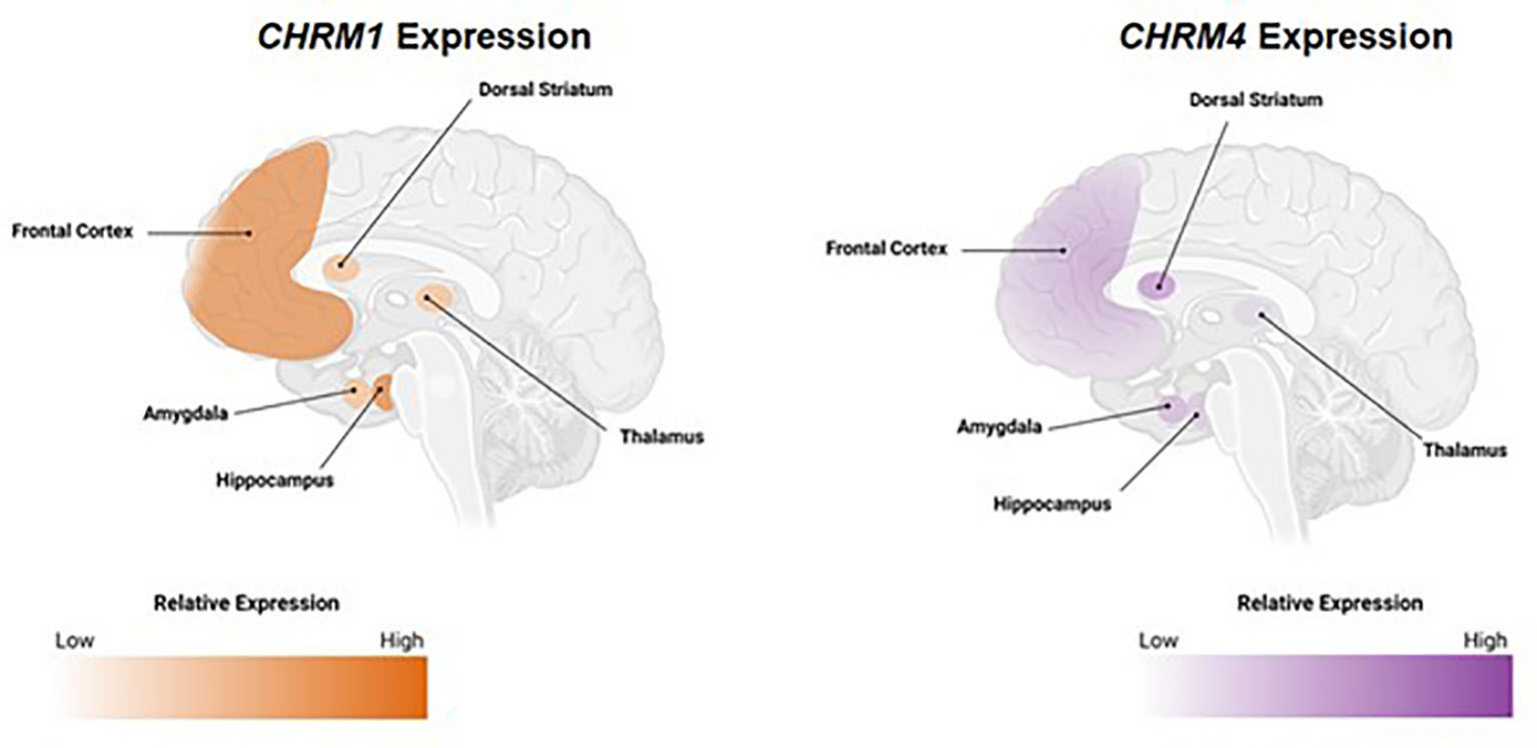
Expression pattern of M_1_ and M_4_ mAChRs in brain regions implicated in cognition. Relative *CHRM1* and *CHRM4* expression from healthy human tissue. All mAChRs can be found in the CNS, but the most prominent subtypes in brain regions implicated in cognition are M_1_ and M_4_ mAChRs. Data retrieved from GTEx Portal on September 6, 2022. Areas depicted represent classical cognition circuits. *CHRM1/4*, cholinergic receptor muscarinic 1/4 genes; CNS; central nervous system; mAChR, muscarinic acetylcholine receptor.

The neural circuit effects of ACh are mediated by two receptor types: nAChRs, which are ligand-gated ion channels, and mAChRs, a five-receptor family of G-protein coupled receptors (GPCRs) (23). Both classes of receptors are expressed in the central (CNS) and peripheral nervous system (PNS). Within the PNS, activation of mAChRs produces end-organ responses that mimic parasympathetic nervous system stimulation (e.g., salivation, urination, and increases in gastric secretion and motility) (24). Within the CNS, mAChRs play important roles in modulating neuronal activity and neurotransmitter release in many brain regions (25). For this review, we focus on M_1_ and M_4_ mAChRs as these receptor subtypes are highly expressed in brain regions implicated in cognitive function (**Figure 1**).

Within the cell membrane, GPCRs bind extracellular substances (e.g., the endogenous ligand, agonists, or antagonists) and transmit signals from these substances to an intracellular molecule called a G-protein (26). Based on signal transduction properties, mAChRs can be grouped into two families. Canonically, M_1_ mAChRs couple to the G_q/11_ family (e.g., excitatory G-proteins), leading to intracellular calcium mobilization and cellular excitability, whereas M_4_ mAChRs preferentially signal through G_i/o_ (e.g., inhibitory G-proteins), leading to inhibition of adenylate cyclase and cellular inhibition.

The pharmacological characterization of therapeutic agents that act on mAChRs has not been a straightforward task due to the high level of conservation at the orthosteric site (site that binds the endogenous ligand) across the mAChR subtypes (25). Therefore, there are very few orthosteric agonists and antagonists that exhibit high selectivity for one subtype over the others. Activation of the orthosteric site can lead to unwanted activation of mAChR subtypes, and as discussed below, this was the shortcoming of many early drug discovery programs. mAChRs also contain an allosteric site that is topographically distinct and less conserved compared with the orthosteric site (27). The common allosteric binding site is located between the second and third transmembrane loops; however, computer modeling studies of allosteric ligands have revealed that allosteric binding to some mAChRs is more complicated. For instance, simulation of molecular dynamics have revealed cryptic allosteric binding pockets in the vicinity of the common allosteric binding site (28). Targeting the allosteric site has afforded the development of selective molecules that are believed to modulate efficacy via actions on critical subtypes while avoiding other subtypes that are believed to contribute to the side effect profile.

### 2.2 Clinical trials with mAChR agonists

Direct acting functional mAChR agonists in the cortex were of interest for many of the early drug discovery programs in AD. This was largely rooted in the etiology and progression of AD, which demonstrated that postsynaptic M_1_ mAChRs were less susceptible to degeneration, making this an attractive target for symptomatic treatment of AD (29). Several compounds were investigated in Phase 2 or Phase 3 clinical trials; however, development of these compounds for the treatment of AD was discontinued due to lack of efficacy, poor side-effect profiles due to stimulation of peripheral mAChRs, and unsuitable pharmacokinetic profiles (30).

Of all the compounds that were investigated for the treatment of cognitive impairment in AD, xanomeline, a dual M_1_/M_4_ mAChR orthosteric agonist initially developed by Eli Lilly & Company advanced the furthest. In one large-scale trial of safety and efficacy in people with AD, xanomeline was associated with enhanced cognition relative to placebo (31). The maximal effect on cognition was evident by 8 weeks of treatment and remained stably improved until the end of the 24-week trial. Notably, the magnitude of the xanomeline-associated cognitive benefit was substantially larger in participants with moderate than in those with mild AD (32). Interestingly, a completely unexpected finding was that xanomeline also improved psychotic-like symptoms. However, the discontinuation rate associated with xanomeline was 58.6% versus 33.3% in those receiving placebo, due primarily to gastrointestinal (GI) side effects attributable to peripheral mAChR agonism.

Based on the unexpected finding that xanomeline improved psychotic-like symptoms, a small proof-of-concept study subsequently evaluated acute schizophrenia among inpatient participants assigned to xanomeline or placebo. Those assigned to xanomeline demonstrated significant improvements in cognitive symptoms (e.g., listing learning, story recall, and delayed memory) as well as positive and negative symptoms (33). However, the peripherally mediated cholinergic side effect profile was again quite poor.

In summary, early clinical drug discovery efforts indicated that stimulation of M_1_ and M_4_ mAChRs in the CNS could produce treatment benefits for cognition and other neuropsychiatric symptoms. However, the therapeutic index for this compound was insufficient, as mAChR stimulation in the CNS was accompanied by peripherally mediated cholinergic side effects. As a result, drug development programs for xanomeline and related compounds were shelved and would remain dormant for many years.

## 3 Development of selective pharmacological agents

**Figure 2:**
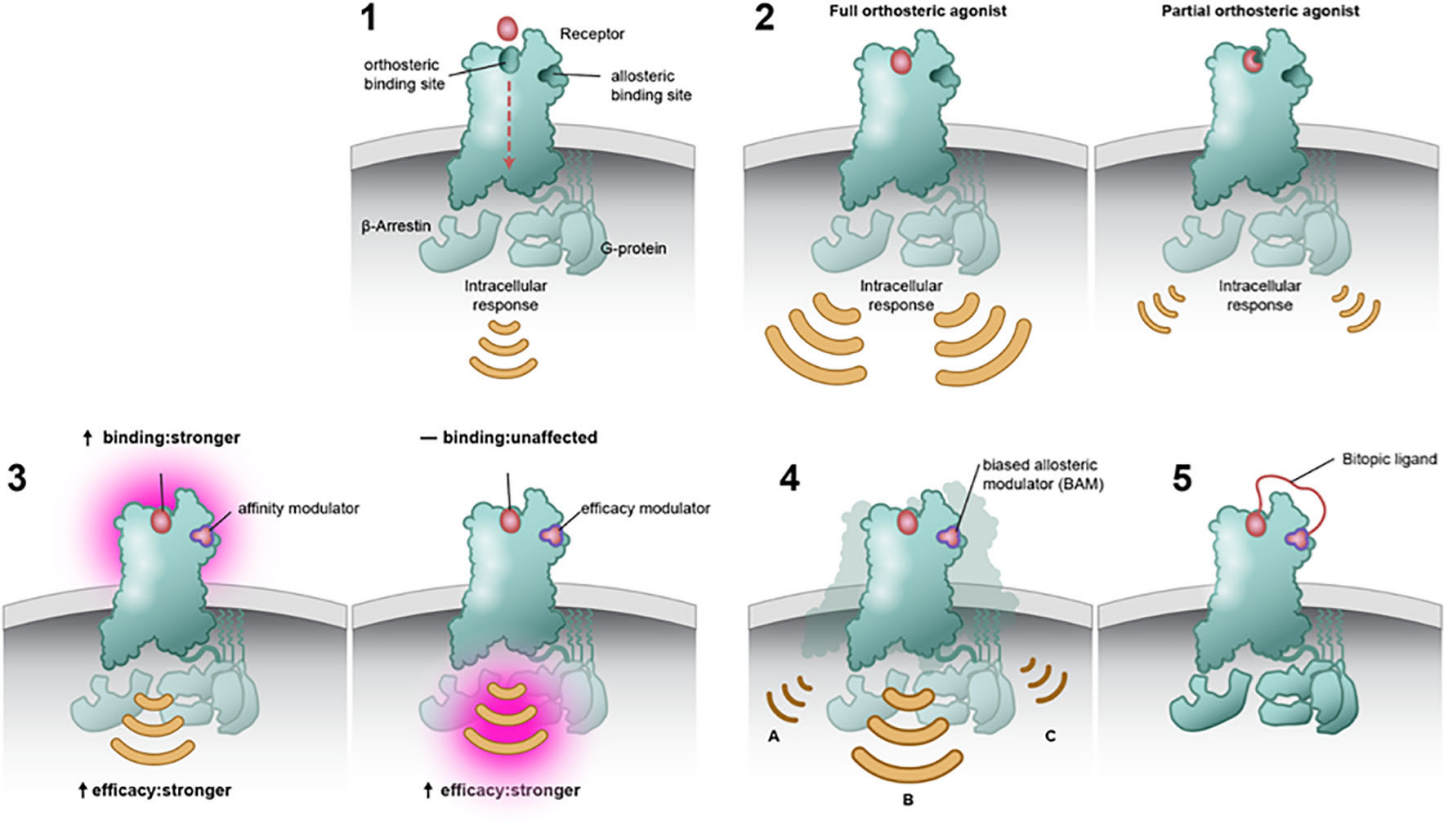
Modes of binding at the orthosteric and/or allosteric site. Muscarinic acetylcholine receptors (mAChRs) contain an orthosteric site, which is predominantly made up of highly conserved residues within the top third of the transmembrane domains. Orthosteric drugs bind at the active site, competing with the natural substrate or ligand. mAChRs also contain a temporal distinct allosteric binding site that is less conserved (panel 1). Full orthosteric agonists bind to and activate the mAChR with the maximum response that an agonist can elicit at the receptor (panel 2a). Partial orthosteric agonists are drugs that bind to and activate a given receptor but have only partial efficacy at the receptor relative to a full agonist. They may also be considered ligands that display both agonistic and antagonistic effects (panel 2b). Allosteric modulators can alter the affinity and efficacy of other substances acting on a receptor. Positive allosteric modulators may increase the response of the receptor by increasing the probability that an agonist will bind to a receptor (i.e., affinity; panel 3a), increasing its ability to activate the receptor (i.e., efficacy; panel 3b), or both. Biased ligands Ligands engage less well-conserved regulatory motifs outside the orthosteric pocket and exert pathway-specific effects on receptor signaling (panel 4). A bitopic ligand makes molecule makes concomitant interaction with both an orthosteric site and an allosteric site upon a single receptor (panel 5).

To overcome intrafamily promiscuity of mAChR orthosteric drugs, efforts have been made to target the allosteric sites of these receptors (25, 34). The classical mAChR allosteric pocket is located just above the orthosteric binding site and is partially formed by extracellular loops, which show greater sequence variation among the receptor subtypes (**Figure 2**, panel 1) (35). Depending on the type of allosteric ligand, binding can result in several changes, such as modifying the affinity of the orthosteric ligand (e.g., strengthening or weakening the binding affinity of ACh; **Figure 2,** panel 3a) (36), changing the intracellular signaling strength (**Figure 2,** panel 3b), or acting as a dualsteric (bitopic) ligand that simultaneously targets the orthosteric and allosteric sites (**Figure 2,** panel 5) (37).

Allosteric modulators have two main classes: positive (PAMs) that increase response to orthosteric agonists and negative (NAMs) that inhibit responses to orthosteric agonists (27, 35). An allosteric modulator would only induce an action when the endogenous neurotransmitter is released (in this case ACh), and its action would be restricted in space and time to those synapses where signaling is currently happening.

Over the past three decades, a structurally diverse array of mAChR selective ligands have been identified and characterized for both M_1_ and M_4_ mAChRs (34, 38). PAMs may differ in their ability to confer receptor signaling, known as signal bias (**Figure 2,** panel 4) (39, 40). That is, activation of mAChRs may cause an effect in all second messenger signaling pathways, whereas a biased molecule would cause an effect in one second messenger pathway over the others. By imparting biased modulation, these allosteric modulators could activate therapeutically relevant signaling pathways while not acting on those pathways thought to be responsible for on-target side effects.

In summary, the unique properties of allosteric ligands necessitate a more comprehensive and nuanced approached to their pharmacological evaluation. Researchers must employ sophisticated techniques to fully understand the therapeutic potential and limitations of allosteric modulators. As discussed below, the development of M_1_ and M_4_ mAChR allosteric modulators has helped to advance our understanding of the roles of these receptors in neural nodes important for cognitive functions.

### 3.1 Modeling cognitive deficits in pre-clinical species

Animal models have played a vital role in investigating the physiological processes and mechanisms associated with M_1_ and M_4_ mAChRs in the neural bases of cognitive function. Most animal models currently being used are chemical- or drug-induced models, which facilitate studies of behavioral effects and neuronal effects. A particularly important animal model focuses on *N*-methyl-d-aspartate (NMDA) receptor dysfunction via administration of NMDA receptor antagonists or genetic mutations (41, 42). NMDA receptor hypofunction is a convergence point for the development and diverse symptoms of schizophrenia, especially cognitive deficits (43, 44). Hypofunction of NMDA receptors causes an imbalance of GABA and Glu neurotransmission in the brain (45), leading to deficits in local neural networks (e.g., hippocampus and prefrontal cortex [PFC]) as well as long-range disconnections between regions of the brain. Reduced excitatory input to NMDA receptors located on GABA inhibitory interneurons in cortical brain regions leads to reduced inhibition of excitatory pyramidal neurons and can cause an excitatory:inhibitory imbalance and perturbed network function that could explain CIAS (16). Use of NMDA receptor antagonists as tool compounds in pre-clinical species has high predictive validity (i.e., high translational potential across people with schizophrenia, healthy volunteers, and pre-clinical studies). For instance, studies have shown altered plasticity in people with schizophrenia (46) and that NMDA receptor antagonists can induce and exacerbate cognitive deficits in clinical populations (41).

In addition to behavioral studies, pre-clinical models can also incorporate electrophysiology and/or microdialysis measures, which provide researchers with a way to “peek under the hood” at what is occurring in the brain. Electrophysiology is the measurement of electrical activity of cells. Neurons in the brain form biochemical synapses with one another that facilitate the transmission of electrical signals between neurons (47). In the brain, synapses can modulate their response to various stimuli through synaptic plasticity; synaptic plasticity is driven by Glu and GABA interactions. Functionally, long-term potentiation (LTP) and long-term depression (LTD) are the two forms of synaptic plasticity (48). LTP is the strengthening of synapses following repeated synaptic activity, whereas LTD is the weakening of synaptic strengths. LTP and LTD are a neural correlate of learning and memory; that is, LTP and LTD are important for suppressing “outdated” memories to allow synapses to be “updated” with current information. Microdialysis is the measurement of neurotransmitter release in brain regions of interest; increases in certain neurotransmitters has been correlated with cognitive function. Changes in ACh and DA content (e.g., release) in hippocampal and frontal brain regions have been associated with cognitive function (49, 50). Although it remains unclear what changes induce the onset of cognitive dysfunction, it is reasonable to hypothesize that alterations to Glu and GABA balance and neurotransmitter disturbances (e.g., ACh and/or DA) could contribute to a disruption in network functioning in schizophrenia.

### 3.2 Behavioral effects of M_1_ and M_4_ mAChR activators: pre-clinical evidence

Pharmacological blockade or genetic deletion of the M_1_ mAChR produces significant learning and memory disturbances in pre-clinical animal models (51–53). M_1_ mAChR activation has been shown to consistently enhance memory consolidation and retrieval in various tasks (e.g., object recognition, spatial learning, and fear conditioning), executive function, attention (54), and cognitive flexibility (55). Additionally, M_1_ mAChR activation can attenuate cognitive deficits induced by NMDA antagonists (56) and genetic mutations in the NMDA receptor (57) (for detailed review on the development of M_1_ mAChR selective ligands see Nguyen et al., 2024 (58)).

In contrast to impairments in memory, learning and attentional accuracy (59) seen in global M_1_ mAChR knockout mice, M_4_ mAChR knockout mice (e.g., mice that have M_1_ or M_4_ mAChR turned off or ‘knocked out’) have robust deficits in the acquisition of both contextual and cue-dependent fear conditioning (60) but not spatial memory (60, 61), suggesting that the role of M_4_ mAChRs may be important for certain types of memory. M_4_ mAChR PAMs have demonstrated pro-cognitive benefits in rodents (60, 62–64) and nonhuman primates (65). Imaging studies have shown that M_4_ mAChR PAM administration can normalize amphetamine-induced changes in hippocampal activity (66). Chronic administration of M_4_ mAChR PAMs can enhance the rate of acquisition (63), an effect absent in mice where the M_4_ mAChR is removed; these findings suggest that M_4_ mAChR PAMs can enhance cognition. Interestingly, the efficacious dose range of M_4_ mAChR PAMs in pre-clinical *in vivo* models for antipsychotic-like activity and cognitive function have nonoverlapping minimal effective dose concentrations (e.g., lower doses are needed to improve cognitive deficits induced by an NMDA antagonist [5.6 mg/kg] versus higher doses to reverse NMDA-induced hyperlocomotor activity [10 mg/kg]) (60). However, additional studies are needed to explore the higher end of the locomotor dose response curve on cognitive function. Overall, although further research is needed to fully understand the role of M_4_ mAChRs in cognition, current evidence suggests their involvement in modulating cognitive processes and possibly influencing emotional memory processes.

In rodents, M_1_ and M_4_ mAChR activators have been found to improve memory in animals that performed poorly at baseline (62), and individual differences in extinction learning, but not acquisition, have been correlated with M_1_ mAChR signaling (67). The findings by Galloway and colleagues (62) align with prior findings which indicate that the benefit of mAChR activation on memory performance is dependent on baseline performance level (68). Future studies are needed to determine whether differences in baseline memory performance is due to individual differences in the level of endogenous ACh or mAChR receptor signaling. It is important to note that these findings (62) and others (69, 70) also support the possibility M_1_ and M_4_ mAChR beyond an optimal range has no beneficial effect and may even be detrimental to average or above average performance, suggesting that the relationship between mAChR stimulation and cognitive function is non-linear and has an inverted U-shaped curve. However, future research is required to explore these findings, particularly within the context of schizophrenia. In summary, dual activation of M_1_ and M_4_ mAChRs can also attenuate deficits in cognition in pharmacological and genetic models (71–73). Taken together, these findings suggest that M_1_ plus M_4_ mAChR activation may have pro-cognitive effects across several cognitive domains.

The impact of M_1_ and M_4_ mAChRs on cognitive function in pre-clinical *in vivo* models is complex and can vary depending on the specific cognitive task, brain region, and experimental conditions. Although both M_1_ and M_4_ mAChRs are implicated in pre-clinical cognitive models, they seem to have distinct roles; their specific contributions in neuronal nodes important for cognitive functioning continue to be actively investigated.

### 3.3 Neuronal actions of M_1_ and M_4_ mAChRs in the hippocampus

M_1_ and M_4_ mAChRs play important roles in the hippocampus, a region of the brain that is critical to cognitive processes. Cholinergic tone within the hippocampus shapes neural circuit function and subsequent behavior (74). Within the hippocampus, M_1_ mAChRs are abundantly expressed across all regions and cell types, and, to a lesser extent, M_4_ mAChRs are expressed in a more regionally specific manner (75). The main function of ACh in the hippocampus is to modulate levels of Glu and GABA (e.g., excitatory and inhibitory transmission) to drive synaptic plasticity as well as neuronal oscillations.

In the hippocampus, ACh facilitates learning and memory through cholinergic induction of neural oscillations (76). mAChRs modulate the excitability and synaptic connectivity of pyramidal neurons located in CA1 and CA3 subregions. That is, transient (short-term) activation of mAChRs within the CA1 region causes an inhibition (engagement of GABAergic interneurons) followed by an excitation (engagement of Glu-containing pyramidal neurons) (77). In contrast to the biphasic nature of mAChR activation in CA1, mAChR activation in the CA3 subregion evokes an excitatory response (e.g., activation of Glu-containing pyramidal neurons) (78).

**Figure 3:**
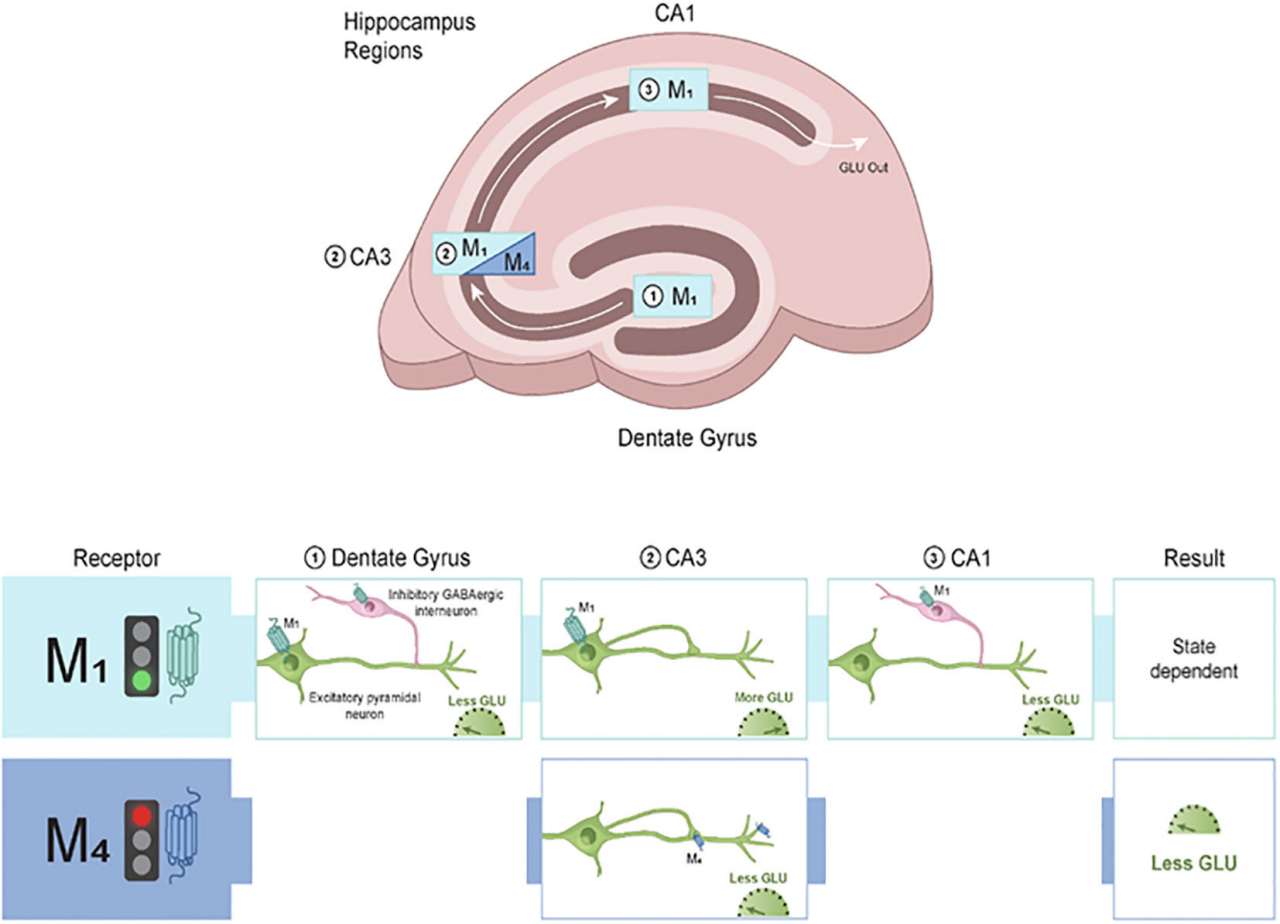
Modulatory role of M_1_ and M_4_ mAChRs in the hippocampus. The hippocampus is a small, curved formation located deep in the temporal lobe of the brain. Neurobiological and functional evidence strongly suggests that the hippocampus is a homologous structure across species. M_1_ mAChRs are distributed across the hippocampal subregions where they can modulate excitatory (Glu) and inhibitory (GABA) neurotransmission. Within the dentate gyrus (DG), activation of M_1_ mAChRs on inhibitory interneurons leads to a reduction of Glu output to CA3. In CA3, activation of M_1_ mAChRs facilitates Glu release, leading to more excitatory neurotransmission into CA1. Within CA1, M_1_ mAChRs are found on inhibitory interneurons and increase GABA input onto excitatory pyramidal neurons. In contrast to the broad distribution pattern of M_1_ mAChRs in the hippocampus, M_4_ mAChRs are primarily located in the CA3 region and modulate excitatory neurotransmission. M_4_ mAChRs synapse back onto themselves and are found on axon terminals, where they gate glutamate release onto CA1. The net output of M_4_ mAChR activation is to decrease excitatory neurotransmission and subsequently lead to decreased Glu output to downstream structures. Taken together, M_1_ and M_4_ mAChRs can fine-tune excitatory and inhibitory balance within the hippocampus that is critical for cognitive function. GABA, gamma-aminobutyric acid; Glu, glutamate; mAChR, muscarinic acetylcholine receptor.

Studies using mice that have mAChR subtypes deleted (e.g., global mAChR knockout mice) suggest that M_1_ is the major mAChR subtype responsible for direct cholinergic modulation of pyramidal neurons within hippocampal circuits (79–82). M_1_ mAChR activation is analogous to that of a switch in that the net effect of turning on the M_1_ mAChR is to facilitate excitatory transmission. For instance, electrophysiology studies have shown reduced or lack of cholinergic modulation of both CA1 and CA3 pyramidal neurons in M_1_ mAChR knockout mice (79, 81). An increase in Glu excitatory neurotransmission leads to LTP, an effect that is mediated via activation of α-amino-3-hydroxy-5-methyl-4-isoxazolepropionic acid (AMPA) receptors (83). This is of particular interest as M_1_ mAChR activation has been reported to facilitate hippocampal memory due to co-communication with AMPA receptors (84, 85). This finding highlights the central role that M_1_ mAChRs play in shaping excitatory synapses involved in learning and memory (**Figure 3**).

GABAergic hippocampal interneurons also modulate firing frequency and neuronal excitability via M_1_ mAChRs (86). In CA1 pyramidal neurons, cholinergic activation of extracellular signal-regulated kinase (ERK) and mitogen-activated protein kinase (MAPK) pathways occur through stimulation of M_1_ mAChRs (87). This finding is important as ERK and MAPK activation is required for the formation and maintenance of LTP (88), a process involving the persistent strengthening of synapses (89). These findings suggest that M_1_ mAChR activation is important for cell-mediated responses.

M_4_ mAChRs also play a role in modulating hippocampal microcircuitry (90). M_4_ mAChR knockout mice treated with the nonselective cholinergic receptor agonist carbachol display a reduced suppression of excitatory postsynaptic potentials between the CA3 and CA1 regions, an effect gated by Glu neurotransmission (79). Thus, ACh can modulate pyramidal neuron excitability directly (via M_1_ mAChR activation; **Figure 3**) as well as through alterations of synaptic transmission between CA3 and CA1 pyramidal neurons (via M_4_ mAChR activation). In line with this, mAChR-induced hippocampal gamma oscillations in CA3 neurons are absent and carbachol-induced depression of transmission at excitatory synapses are blunted in M_1_ and M_4_ mAChR knockout mice, respectively (91, 92).

The correct balance of inhibitory and excitatory neurotransmission in the hippocampus is a key feature of learning and memory processes. A disruption in this balance can result in cognitive impairments (93, 94). M_1_ and M_4_ mAChRs can exert modulatory effects on synaptic plasticity processes within the hippocampus to ensure appropriate filtering of information and changes in downstream areas (**Table 1**) (95). This perspective seems consistent with clinical data that suggest an increased signal-to-noise ratio within the hippocampus can improve memory encoding accuracy (96) via preserving the excitatory and inhibitory balance and gating activity in hippocampal subregions.

**Table 1:** Summary of activation of M_1_ and M_4_ mAChRs on neuronal circuits.

	M_1_ mAChR activation	M_4_ mAChR activation	Dual M_1_/M_4_ mAChR activation
Frontal cortex	• Layer-specific effects on glutamate/GABA neurotransmission • Increases ACh and DA release from midbrain areas	• Reduction of glutamate transmission within layer V	• Layer-specific effects on glutamate/GABA neurotransmission • Increases ACh and DA release from midbrain areas • Induction of immediate early gene expression
Hippocampus	• Region-specific effects on glutamate/GABA neurotransmission • Increase in immediate early gene expression • Normalization of gamma oscillations	• Reduction of glutamate transmission within CA3 • Reduction of excitatory drive between CA3 and CA1 • Normalization of gamma oscillations	• Region-specific effects on glutamate/GABA neurotransmission • Increase in immediate early gene expression • Normalization of gamma oscillations

ACh, acetylcholine; DA, dopamine; GABA, gamma-aminobutyric acid; mAChR, muscarinic acetylcholine receptor.

### 3.4 Neuronal actions of M_1_ and M_4_ mAChRs in the frontal cortex

The functional microcircuitry of the PFC is shaped by cholinergic input from midbrain regions (97). Within the PFC, cholinergic tone is a crucial component of cognitive function, and cholinergic input acts as a gatekeeper to modulate synaptic plasticity and tone various other neurotransmitter systems. Similar to its actions within the hippocampus, ACh activity within the PFC is primarily modulated via M_1_ and M_4_ mAChRs. As discussed below, the location of these receptors within the PFC allows them to exert a variety of effects on cognitive function; thus, understanding the role of M_1_ and M_4_ mAChRs in shaping cognitive functions is crucial for developing potential therapeutic strategies for cognitive impairment.

**Figure 4:**
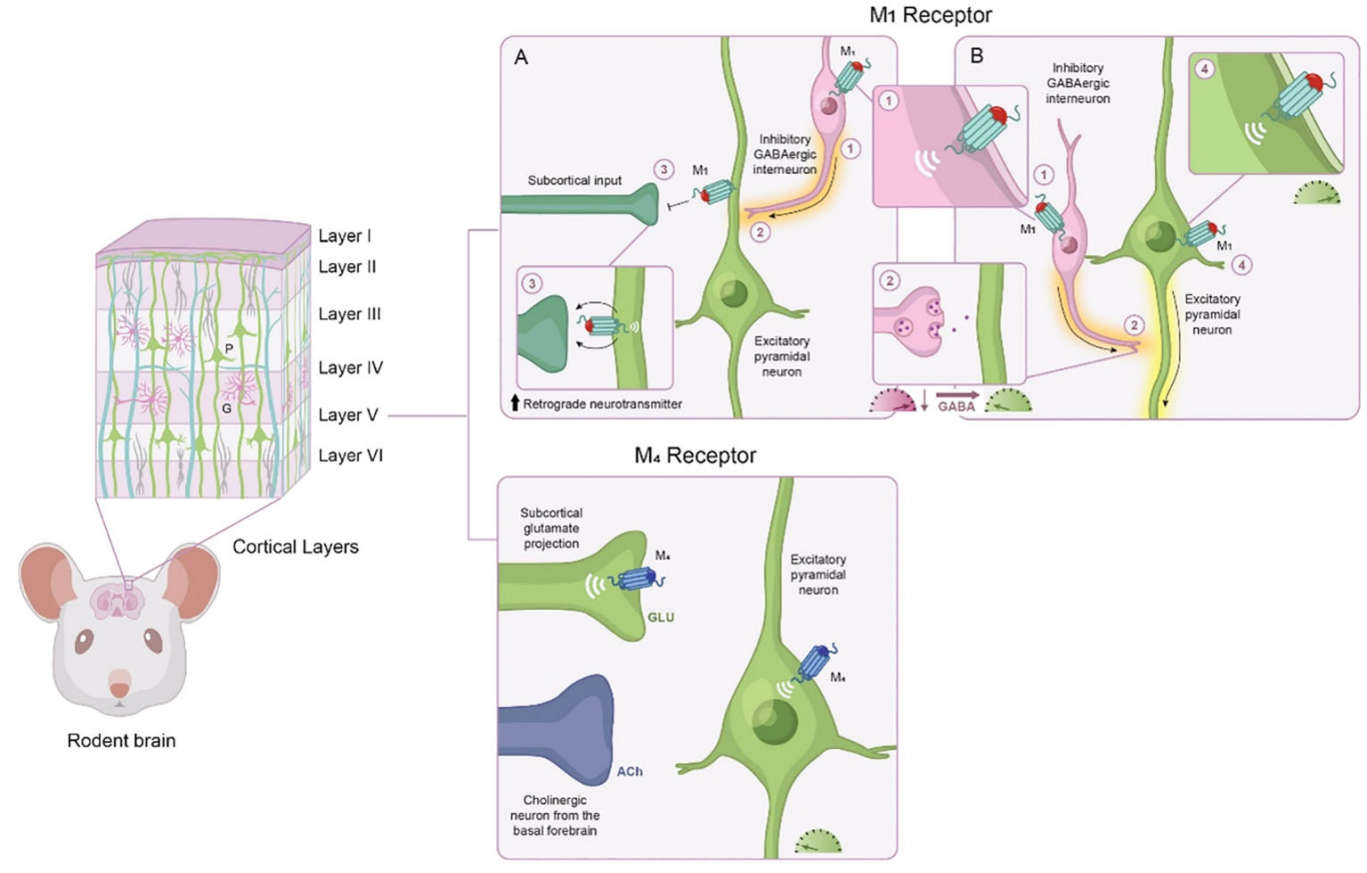
Modulatory role of M_1_ and M_4_ mAChR activation in the frontal cortex. The frontal cortex has some degree of laminar organization, with different layers composed of neurons with distinct connectivity patterns and molecular profiles. Based on cytoarchitectural different, the rodent frontal cortex is classified into four distinct neuroanatomical subregions along a dorsal to ventral axis. The frontal cortex neural networks consist predominately of excitatory pyramidal neurons (green) and inhibitory GABAergic interneurons (purple). Based on their physiological and molecular properties, interneurons can be divided into several subpopulations. M_1_ mAChRs are positioned to modulate a wide variety of neuronal activity; the actions of M_1_ mAChR activation depend on which cell type M_1_ mAChRs are expressed, the location of M_1_ mAChRs, and the receiving neuron. **(A)** M_1_ mAChR activation induces a form of long-term depression of glutamatergic inputs from subcortical areas, such as the ventral hippocampus, via activation of retrograde neurotransmitters. **(B)** M_1_ mAChR activation also increases the excitability of pyramidal neurons (i.e., more glutamate release to downstream structures) and GABAergic interneurons (i.e., increased inhibition onto glutamatergic neurons, meaning less glutamate output to downstream structures). Activation of M_1_ mAChRs via GABAergic interneurons also can increase gamma oscillation synchrony in the cortex. Within layer V, M_4_ mAChRs are located on subcortical glutamate projections into the prefrontal cortex where they can modulate glutamate release within layer V. M_4_ mAChRs are also found on Layer V principal output neurons of the prefrontal cortex, where they contribute to hyperpolarization. GABA, gamma-aminobutyric acid; Glu, glutamate; mAChR, muscarinic acetylcholine receptor.

Transient ACh release onto pyramidal neurons causes a depolarization of layer 5 pyramidal neurons via M_1_ mAChR activation, causing them to fire Glu to downstream structures, whereas constant presence of ACh does the opposite (98). In this sense, M_1_ mAChR activation gates neuronal activity through two approaches: sustained tonic stimulation, which leads to reduced excitability in output structures, versus transient phasic stimulation, which leads to increased excitability in output structures (**Figure 4**). These two modes represent a form of communication to sharpen the signal-to-noise ratio within the local microcircuits of the PFC. This is particularly important because in states characterized by NMDA hypofunction, like schizophrenia, the signal-to-noise ratio is disrupted (99). M_1_ mAChR activation can restore burst activity and accentuate the signal transmission efficiency of PFC pyramidal neurons in NMDA hypofunction states (100).

Pre-clinical *in vivo* and *ex vivo* evidence suggests that phasic and tonic ACh release can occur concurrently during performance of cognitive tasks (101). Further, since M_1_ mAChRs are coupled to diverse signaling pathways, both excitatory and inhibitory responses may co-occur in the same neuron (102). The weight and direction of the response is an important feature underlying learning and memory, as alterations in behavior (attentional switching, reaction time, etc.) are caused by changes in neuronal firing. It is also important to note that the activity of M_1_ mAChRs may be layer specific. For instance, differences in cholinergic responsiveness of pyramidal neurons have been reported between layers 2/3 and layer 5, an effect most likely attributed to calcium-activated potassium channels (103).

Although the M_4_ mAChR has not been traditionally associated with actions in the PFC, emerging data has shed light on the modulatory role of M_4_ mAChRs within cognition microcircuits. M_4_ mAChR activation has been reported to decrease excitatory Glu transmission at corticostriatal synapses, resulting in LTD (104), which gates downstream activity and therefore shapes contextual representations (**Figure 4**). The output gating dynamic for working memory depends on interactions between the cortex and the striatum (105). The excitatory drive from corticostriatal glutamatergic afferents activates striatal neurons, which, in turn, alters the activity of neurons throughout the entire basal ganglia. Neuroimaging studies demonstrate that schizophrenia is associated with aberrant connectivity of the corticostriatal network (106). Although future studies are needed to investigate the relationship between abnormal connectivity and cognitive function, these findings suggest that M_4_ mAChR activation shapes corticostriatal network activity and modifying M_4_ mAChR activity could have beneficial effects on cognitive processes dependent on this network.

In addition to shaping plasticity, M_1_ and M_4_ mAChR activation in the PFC has been shown to facilitate ACh neurotransmission (66, 72, 107, 108). In fact, this idea was the driving factor for early drug development programs targeting the cholinergic system in AD; that is, an increase in ACh in the PFC was expected to improve cognitive function (30). It is hypothesized that ACh enhances the encoding of memory by facilitating feedforward, top-down output.

The PFC has reciprocal connectivity with several neuromodulatory systems, including the midbrain DA system (109). In the rodent brain, ventral tegmental area (VTA) DA neurons send sparse projections to frontal regions (110). It is important to note that there is considerable variation of DA integration of the PFC across mammalian species. Pre-clinical literature supports the idea that mesocortical DA is functionally distinct from mesolimbic DA (for detailed review, see (111). Cholinergic neurotransmission can orchestrate DA dynamics within the PFC. For instance, dual M_1_/M_4_ mAChR activation has been shown to increase DA release in the PFC in rodent models (72, 108). This increase in PFC DA release may be one of the ways that dual M_1_/M_4_ mAChR activation facilitates elements of cognitive functioning.

Postsynaptically, DA exerts its actions in the PFC via D_1_ DA receptor activation (112, 113). Hypofunction of the D_1_ DA receptor pathway may underlie cognitive dysfunction (114, 115). Within the PFC, signal transduction crosstalk between M_1_ mAChRs and D_1_ DA receptors within pyramidal cell dendrites has been reported (116). This interaction may be relevant for optimizing the level of D_1_ DA receptor stimulation that is required for working memory performance (117, 118). Previously, D_1_ DA and mAChR interplay has been characterized in the striatum, where activation of M_4_ mAChRs inhibits D_1_ DA receptor second messengers (119, 120). These findings suggest that a dual M_1_/M_4_ mAChR agonist, like xanomeline, may be beneficial in restoring aberrant D_1_ DA receptor signaling in the striatum (associated with antipsychotic-like activity) via M_4_ mAChR activation and the frontal cortex via M_1_ mAChR activation (associated with cognitive improvement).

Taken together these findings suggest that M_1_ and M_4_ mAChR activation can shape neuronal activity of the PFC in multiple ways. The interplay between M_1_ and M_4_ mAChRs can modulate the balance of excitatory and inhibitory signaling in neural circuits, ultimately shaping neuronal activity and cognitive processes. It is important to note that the roles of M_1_ and M_4_ mAChRs in the PFC and hippocampus can be complex and context dependent (**Table 2**).

**Table 2:** Active clinical and pre-clinical mAChR programs for schizophrenia.

Clinical					
Company	Compound name	Target	Mode of action	Indication	Stage of development
Karuna Therapeutics (recently acquired by BMS)	KarXT	M_1_/M_4_	Muscarinic Agonists + peripherally restricted mAChR antagonist	SCZ and AD psychosis	Phase 3
AbbVie (previously Cerevel Therapeutics)	Emraclidine	M_4_	PAM	SCZ	Phase 2
Neurocrine Biosciences	NBI-1117568	M_4_	Agonist	SCZ	Phase 2
Anavex Life Sciences	ANAVEX3-71	Sigma 1/M_1_	Agonist/PAM	SCZ and AD cognition	Phase 2
Neurocrine Biosciences	NBI-1117570	M_1_/M_4_	Agonist	SCZ	Phase 1
MapLight Therapeutics	ML-007	M_1_/M_4_	Muscarinic Agonists + peripherally restricted mAChR antagonist	SCZ and AD	Phase 1
Neumora Therapeutics	NMRA-266	M_4_	PAM	SCZ	Phase 1
Pre-clinical					
Company	Compound Name	Target	Mode of Action	Indication	
Addex Therapeutics	–	M_4_	PAM	SCZ	
NeuroSolis	NSX-0527	M_1_/M_4_	Agonist	SCZ and AD	
	NSX-0559				
NeuShen Therapeutics	NS-136	M_4_	PAM	SCZ	
Cerevel Therapeutics	–	M_4_	Agonist	SCZ and AD	
Neurocrine Biosciences	NBI-1117569	M_4_	Agonist	SCZ	
Suven Life Sciences	SUVN-17016031	M_1_	PAM	PD dementia	
	SUVN-L8203032	M_4_	PAM	SCZ	
	SUVN-16107	M_1_	PAM	Cognition	
	SUVNI-1307014	M_1_	PAM	AD	
Asceneuron	–	M_1_	Agonist	Frontotemporal dementia	

AD, Alzheimer’s disease; mAChR, muscarinic acetylcholine receptor; PAM, positive allosteric modulator; PD, Parkinson’s disease; SCZ, schizophrenia.

### 3.5 Non-neuronal actions of M_1_ and M_4_ mAChRs in memory

There is considerable support for the role of cytokine release from microglia in the modulation of memory. Administration of inflammatory cytokines causes deficits in spatial memory in pre-clinical behavioral models (121); an altered immune profile has been reported in people with mild cognitive impairment (122), and associations between cytokine levels and cognition in people with chronic and first-episode schizophrenia has been reported (123, 124). In pre-clinical models, the dual M_1_/M_4_ mAChR agonist xanomeline has been shown to suppress excessive pro-inflammatory cytokine responses (125), and recent data suggests that M_4_ mAChR activation alone can reduce pro-inflammatory cytokines (126); however, additional work is needed. Additional trials in clinical populations are needed to confirm non-neuronal actions of mAChR activators.

## 4 Clinical neuroscience support for mAChR drug development in schizophrenia

In the past 5 years, there has been a resurgence of interest in the mACh system for the treatment of CIAS. This renewed interest has been catalyzed by a much deeper neuroscience-based understanding of the mAChRs in cognitive function based on evidence from *in vitro* and *in vivo* pre-clinical studies. Additionally, several lines of clinical research evidence have implicated cholinergic functioning in the pathophysiology of cognitive impairment in schizophrenia.

- Postmortem studies demonstrate reductions of M_1_ mAChRs in the dorsolateral PFC and M_4_ mAChRs in the hippocampus, with relative sparing of M_2_ and M_3_ mAChRs, in schizophrenia (127).
  - In medication free subjects with psychosis, the reduction in M_1_ mAChRs in the dorsolateral PFC and hippocampus was shown to be related to overall performance in verbal learning and delay in recognition of verbal memory (128).
- Molecular neuroimaging in medication-free individuals with early psychosis provide *in vivo* evidence of reduced M_1_/M_4_ mAChRs. A decrease in M_1_/M_4_ mAChRs provides preliminary *in vivo* support for a disbalance in M_1_/M_4_ mAChR expression in schizophrenia that might directly impact clinical outcomes (129).
- Exposure to medications with higher anticholinergic (e.g., mAChR antagonist pharmacology) burden is associated with impaired cognition in people with schizophrenia (130), although this is a wide-ranging effect across conditions. Patients exposed to high anticholinergic burden have lower brain activity in the frontoparietal network, a flexible hub for cognitive control, and lower performance during working memory tasks as compared with patients with low anticholinergic medication exposure (131).
  - The cognition impairing effects observed with mAChR antagonist pharmacology are due to activation of CNS mAChRs (132). For instance, a prior study found that patients with schizophrenia on maintenance treatment plus centrally active anticholinergic antiparkinsonian drugs (e.g, benztropine) had significant impairment on free recall compared to the placebo group.
- There is some evidence that the mechanistic uniqueness of the antipsychotic clozapine may be due to its active metabolite *N*-desmethylclozapine (NDMC), a partial M_1_ mAChR agonist. NMDC increases cortical ACh and DA release (133). In clinical populations, lower clozapine:NDMC ratios are associated with improvements in working memory and executive function, whereas higher ratios are associated with cognitive deficits (134).

As discussed below, there are data currently available from one mAChR agent, KarXT, in relation to cognitive function in individuals with schizophrenia, and several other mAChR agents are currently in clinical development.

### 4.1 KarXT clinical development program

Despite xanomeline’s promising efficacy profile described above (see Section 2.2), the development of xanomeline was discontinued because of significant levels of cholinergic adverse events (AEs), namely nausea, vomiting, diarrhea, excessive sweating, and salivary hypersecretion (31, 33), due to stimulation of peripheral M_1_, M_2_, and M_3_ mAChRs. KarXT is a combined formulation of two drugs, xanomeline and trospium chloride, that was designed to mitigate the peripheral mAChR side effects observed with xanomeline. Trospium is a quaternary ammonium compound with a permanent cationic charge that limits its ability to meaningfully cross the blood-brain barrier (135). Thus, trospium competes with xanomeline for binding at peripheral, but not central, mAChRs, thereby reducing the negative mAChR side effects of xanomeline without impacting the potential therapeutic effects of xanomeline in the brain (136).

KarXT was developed as a monotherapy for the treatment of schizophrenia in adults. Across three pivotal, 5-week, Phase 2 and Phase 3 trials (NCT03697252, NCT04659161, and NCT04738123) with acutely symptomatic inpatient participants, KarXT demonstrated a significant improvement compared with placebo on the Positive and Negative Syndrome Scale (PANSS) total score primary efficacy endpoint, and results for secondary endpoints (PANSS positive and negative subscale scores) were typically significant and reproducible (137, 138). KarXT was generally well tolerated and not associated with many of the AEs typically associated with current antipsychotics. These trials supported the submission of a New Drug Application in September 2023 for KarXT, which has the potential to be the first of a new class of medicines based on activating mAChRs, as opposed to the D_2_ DA receptor blocking activity associated with current antipsychotic medications.

Based on the strong mechanistic link between M_1_ and M_4_ mAChR stimulation and cognition, all three pivotal KarXT trials evaluated cognition as an exploratory outcome. In the Phase 2 trial, participants completed an abbreviated computerized battery at baseline and end of treatment. Sample-wide, cognitive improvement was numerically but not statistically greater with KarXT than with placebo. However, a *post hoc* analysis of participants with clinically significant cognitive impairment at baseline, defined as performing at least 1 SD below healthy normative standards, indicated that those treated with KarXT showed a robust, significant cognitive improvement compared with placebo (Cohen’s *d* = 0.50). Further, cognitive improvements were minimally associated with PANSS total symptom changes (139). Prespecified analyses of the exploratory cognitive endpoint in the combined sample from the Phase 3 trials (completed at baseline, week 3, and end of treatment) replicated these findings. There was, again, no significant treatment effect across the entire sample (*N* = 307); however, in the cognitively impaired subgroup (*n* = 137), participants taking KarXT showed significantly greater improvement in cognition compared with placebo (Cohen’s *d* = 0.54) (140). The improvement in cognition was fully independent of changes in PANSS total, positive subscale, and negative subscale scores.

Collectively, the KarXT clinical trials reflect the first time a monotherapy for the treatment of schizophrenia has shown a replicable cognitive benefit across Phase 2 and Phase 3 trials. Although the KarXT effect on cognition did not appear to be secondary to symptom changes (i.e., it was not “pseudo-specific”), the MATRICS CIAS trial guidelines, which focus on testing adjunctive or cotreatment agents in stabilized people with schizophrenia, recommend that assessment of pro-cognitive effects for broad-spectrum agents should also utilize people who are stable. Thus, although these initial findings are encouraging, replication in a longer, well-controlled trial with clinically stable people is needed to fully characterize the potential benefit of KarXT for CIAS.

### 4.2 Other mAChR compounds in development for schizophrenia

At the time of this review several orthosteric and allosteric mAChR compounds have been identified as having potential antipsychotic activity and cognition-enhancing properties in clinical populations and pre-clinical drug development pipelines (**Table 2**).

#### 4.2.1 Emraclidine

Emraclidine (CVL-231) is an M_4_ mAChR PAM (e.g., it selectively activates M_4_ mAChRs) currently under development by AbbVie (previously Cerevel Therapeutics). In a Phase 1b trial (NCT04136873), emraclidine demonstrated a clinically meaningful and statistically significant improvement in PANSS total score at week 6 in participants with schizophrenia compared with placebo (141). At present, three Phase 3 clinical trials are ongoing to confirm the efficacy, safety, and tolerability of emraclidine.

#### 4.2.2 ML-007, ANAVEX3-71, and Neurocrine

ML-007, currently under development by MapLight Therapeutics, is a dual M_1_/M_4_ mAChR agonist paired with a peripherally restricted mAChR antagonist. ML-007 has completed three Phase 1 trials (one trial with an extended-release formulation [ML-007C-MA]) in healthy volunteers. Phase 2 trials with the extended-release formulation are anticipated to begin later this year.

There are currently two other clinical development programs harnessing the potential of mAChR activation for schizophrenia, namely CIAS. Earlier this year, Anavex Life Sciences announced it is recruiting for a Phase 2 trial with ANAVEX3-71, a dual sigma 1 agonist/M_1_ mAChR PAM, in participants with schizophrenia (NCT06245213). This trial aims to assess the benefit of ANAVEX3-71 on positive, negative, and cognitive symptoms of schizophrenia. Originally developed for AD, ANAVEX3-71 has demonstrated efficacy in animal models of cognitive impairment (e.g., transgenic disease models (142) and deficit states (143)). Additionally, although no data are currently available, Neurocrine Biosciences has announced an interest in M_1_ and M_4_ mAChR activators for treating the cognitive symptoms of schizophrenia.

There has been a resurgence of interest in the mAChR system for various neurological and neuropsychiatry disorders, including CIAS. Notably, a handful of other mAChR agents, with various pharmacology flavors, are being investigated in AD (for detailed review, see Johnson et al., 2022 (34) and Felder et al., 2018 (144)). Continued research in this area is needed to deepen our knowledge and lead to the development of innovative therapies and, as such, many novel mAChR agents are in early development (**Table 2**).

## 5 Future directions and conclusions

Schizophrenia is characterized by an array of symptoms that vary in their response to treatment. Even when positive symptoms are effectively managed, negative and cognitive symptoms frequently persist. It is widely recognized that available antipsychotic medications inadequately treat these functionally disabling symptoms (145, 146). Thus, effective new treatments for negative and cognitive symptom domains that target different neural pathways are urgently needed.

Nearly three decades have passed since the cholinergic hypothesis first motivated early drug discovery efforts to become an approach toward the improvement of cognitive systems in AD. Since that time, our understanding of the regional expression and functional roles of M_1_ and M_4_ mAChRs in cognitive circuits has grown dramatically. Similar to what was observed in AD, cognitive functioning in people with schizophrenia was improved by treatment with the dual M_1_/M_4_ mAChR preferring agonist xanomeline (33). As a result of these findings, several novel mAChR therapeutic strategies have emerged, including combining xanomeline with the peripherally restricted pan-mAChR antagonist trospium (KarXT) to reduce peripheral cholinergic side effects as well as developing more subtype selective orthosteric and allosteric agents targeting either M_1_ or M_4_ mAChRs. Recent pivotal placebo-controlled clinical trials demonstrate that KarXT is an effective, well-tolerated monotherapy for positive symptoms, and possibly for cognitive impairment as well. However, future trials will be required to confirm the potential efficacy of KarXT in treating cognitive symptoms. Several other compounds that target M_1_ and/or M_4_ mAChRs more selectively are in earlier stages of clinical development. This emerging new class of mAChR therapies may provide long-awaited breakthroughs in the treatment of CIAS.
